# Trends in Nonfatal Falls and Fall-Related Injuries Among Adults Aged ≥65 Years — United States, 2012–2018

**DOI:** 10.15585/mmwr.mm6927a5

**Published:** 2020-07-10

**Authors:** Briana Moreland, Ramakrishna Kakara, Ankita Henry

**Affiliations:** ^1^Synergy America, Inc. Atlanta, Georgia; ^2^Oak Ridge Institute of Science and Education, Oak Ridge, Tennessee; ^3^Division of Injury Prevention, National Center for Injury Prevention and Control, CDC.

Falls are the leading cause of injury among adults aged ≥65 years (older adults) in the United States. In 2018, an estimated 3 million emergency department visits, more than 950,000 hospitalizations or transfers to another facility (e.g., trauma center), and approximately 32,000 deaths resulted from fall-related injuries among older adults.* Deaths from falls are increasing, with the largest increases occurring among persons aged ≥85 years (*1*). To describe the percentages and rates of nonfatal falls by age group and demographic characteristics and trends in falls and fall-related injuries over time, data were analyzed from the 2018 Behavioral Risk Factor Surveillance System (BRFSS) and were compared with data from 2012, 2014, and 2016. In 2018, 27.5% of older adults reported falling at least once in the past year, and 10.2% reported an injury from a fall in the past year. The percentages of older adults reporting a fall increased between 2012 and 2016 and decreased slightly between 2016 and 2018. Falls are preventable, and health care providers can help their older patients reduce their risk for falls. Screening older patients for fall risk, assessing modifiable risk factors (e.g., use of psychoactive medications or poor gait and balance), and recommending interventions to reduce this risk (e.g., medication management or referral to physical therapy) can prevent older adult falls (https://www.cdc.gov/steadi).

BRFSS is a landline and mobile telephone survey conducted annually in all 50 U.S. states, the District of Columbia (DC), and U.S. territories, with a median response rate of 49.9% in 2018. The survey collects information on health-related behavioral risk factors and chronic conditions among noninstitutionalized U.S. adults aged ≥18 years.^†^ Information on falls and fall-related injuries is recorded every 2 years from adults aged ≥45 years by asking “In the past 12 months, how many times have you fallen?” If the response was one or more times, the respondent was asked “How many of these falls caused an injury? By an injury, we mean the fall caused you to limit your regular activities for at least a day or to go see a doctor.” Responses to each of these questions ranged from 0 to 76 falls or fall-related injuries. Rates were calculated as the average number of falls and fall-related injuries per 1,000 older adults. Both questions were dichotomized to calculate the percentage of older adults who reported having at least one fall or fall-related injury.

Using 2018 BRFSS data, percentages and rates were calculated by age group for demographic (sex and race/ethnicity) and geographic (urban/rural status) characteristics. Functional characteristics (blind/difficulty seeing, difficulty dressing/bathing, difficulty walking/climbing stairs, difficulty doing errands alone, and difficulty concentrating/making decisions) also were compared, as were self-reported health status and data on taking part in any physical activity/exercise in the past month. Analysis was restricted to respondents aged ≥65 years residing in the 50 states and DC. Any respondents with missing values or responses of “Don’t know/Not sure” or “Refused” for falls or fall-related injuries were excluded. Overall, 4.8% of respondents were excluded from the analysis of falls, leaving 142,834; and 4.9% were excluded from the analysis of fall-related injuries, leaving 142,591. Two-sample t-tests were used to compare percentages across characteristics. Linear trend tests were conducted for age group and self-reported health status. BRFSS data from 2012, 2014, 2016, and 2018 were used to examine trends in the percentages of adults aged ≥65 years who had fallen or had a fall-related injury and rates of falls overall and by age group. Polynomial linear regression was used to assess linearity of trends (*2*). Where nonlinear trends were detected, two-sample t-tests with Bonferroni adjustments for multiple comparisons were performed to determine differences between years (*2*). Because the BRFSS questions about falls differed in three states (Michigan, Oregon, and Wisconsin) for 2012, compared with other years, the trend analysis was limited to 47 states and DC. All results presented are weighted to represent the U.S. population. Analysis was conducted using SAS-callable SUDAAN (version 11; RTI International) to account for the complex survey design.

In 2018, 27.5% of adults aged ≥65 years reported at least one fall in the past year (Table 1), and 10.2 % of adults aged ≥65 years reported at least one fall-related injury (Table 2). In the preceding year, an average of 714 falls (Table 1) and an average of 170 fall-related injuries were reported per 1,000 older adults (Table 2), or approximately 35.6 million falls and 8.4 million fall-related injuries. The percentage of adults aged ≥65 years reporting a fall or a fall-related injury increased with age (p<0.001). Among adults aged ≥85 years, 33.8% reported a fall (Table 1) and 13.9% reported a fall-related injury (Table 2). Overall, a higher percentage of women reported at least one fall (29.1%; p<0.001) or fall-related injury (11.9%; p<0.001) than did men in the past year (25.5% reported a fall and 7.9% reported a fall-related injury). However, when stratified by age group, the percentages of adults aged ≥85 years reporting a fall (32.8% of women and 35.7% of men; p = 0.184) or fall-related injury (14.3% of women and 13.4% of men; p = 0.553) did not differ significantly by sex. A lower percentage of blacks (22.5%; p<0.001) and Asian/Pacific Islanders (15.6%; p<0.001) reported a fall than did whites (28.3%) (Table 1), and a higher percentage of American Indian/Alaska Natives (15.2%) reported a fall-related injury than did whites (10.2%; p = 0.008) (Table 2). The percentages of older adults reporting a fall decreased as health status improved (p<0.001) (Table 1). Overall, a higher percentage of older adults living in rural areas (29.5%) reported a fall compared with those living in urban areas (27.0%; p<0.001); however, when stratified by age group, this was only true for persons aged 65–74 years (Table 1). Regardless of age group, older adults reporting difficulties with functional abilities reported a higher percentage of falls and fall-related injuries than did those without these difficulties (p<0.001). A lower percentage of older adults who reported any physical activity in the past month reported a fall (24.9%) compared with those who did not report physical activity (33.1%; p<0.001), regardless of age group.

**TABLE 1:** Number of falls, percentages of adults reporting a fall, and rates* of self-reported falls in the past year among adults aged ≥65 years, by age group and selected characteristics (unweighted n = 142,834) — Behavioral Risk Factor Surveillance System, United States, 2018

Age group/Characteristic	No.^†^ reporting a fall	% (95% CI)^§^	Rate* of falls (95% CI)
**Total (all aged ≥65 years)**			
**Overall**	**13,685,662**	**27.5 (26.9–28.0)**	**714 (689–739)**
**Sex**			
Male	5,629,838	25.5 (24.6–26.3)	735 (694–775)
Female	8,026,432	29.1 (28.3–29.8)	695 (664–727)
**Race/Ethnicity** ^¶^			
White	10,898,569	28.3 (27.8–28.9)	738 (710–765)
Black	4,260,153	22.5 (20.4–24.7)	526 (455–597)
American Indian/Alaska Native	325,910	32.2 (27.3–37.5)	1,169 (845–1494)
Asian/Pacific Islander	237,985	15.6 (10.9–21.8)	250 (167–334)
Hispanic	1,039,618	28.1 (24.7–31.7)	677 (555–799)
Multiple/Other	193,208	29.6 (26.3–33.2)	1,333 (859–1,807)
**Geography**			
Urban	11,024,283	27.0 (26.4–27.7)	682 (653–710)
Rural	2,661,031	29.5 (28.5–30.4)	858 (805–910)
**Self-reported health**			
Excellent	974,558	16.4 (15.0–18.0)	288 (254–323)
Very good	3,201,506	21.9 (21.1–22.8)	420 (393–446)
Good	4,423,458	26.6 (25.6–27.7)	615 (573–657)
Fair	3,246,406	36.8 (35.2–38.3)	1,102 (1,030–1,173)
Poor	1,789,371	48.1 (45.8–50.5)	2,057 (1,872–2,242)
**Functional characteristics**			
Blind/Difficulty seeing			
Yes	1,611,580	42.1 (39.5–44.9)	1,500 (1,343–1,658)
No	12,013,980	26.2 (25.6–26.8)	646 (622–670)
Difficulty concentrating			
Yes	2,398,304	48.5 (46.1–50.9)	1,798 (1,660–1,936)
No	11,133,899	25.0 (24.5–25.6)	584 (562–607)
Difficulty walking/climbing stairs			
Yes	6,218,999	46.3 (45.0–47.6)	1,562 (1,488–1,637)
No	7,386,736	20.4 (19.9–21.0)	397 (377–418)
Difficulty performing errands alone			
Yes	2,578,010	53.0 (50.6–55.3)	1,994 (1,845–2,142)
No	11,017,965	24.6 (24.1–25.2)	573 (550–595)
Difficulty dressing/bathing			
Yes	1,584,599	58.7 (55.6–61.7)	2,496 (2,258–2,735)
No	12,068,592	25.6 (25.1–26.2)	610 (588–633)
Any physical activity in past month			
Yes	8,431,996	24.9 (24.2–25.5)	583 (555–612)
No	5,227,220	33.1 (32.0–34.2)	989 (938–1,040)
**65–74 years**			
**Overall**	**7,619,118**	**25.9 (25.2–26.6)**	**700 (668–733)**
**Sex**			
Male	3,224,096	23.3 (22.2–24.4)	702 (654–750)
Female	4,378,780	28.2 (27.2–29.2)	698 (654–741)
**Race/Ethnicity** ^¶^			
White	5,832,525	26.3 (25.6–27.0)	721 (685–758)
Black	588,611	21.7 (19.4–24.1)	537 (437–638)
American Indian/Alaska Native	72,207	33.9 (27.7–40.7)	1,323 (856–1,790)
Asian/Pacific Islander	182,037	17.8 (11.6–26.4)	269 (160–378)
Hispanic	685,669	28.5 (24.2–33.3)	660 (544–776)
Multiple/Other	112,714	28.2 (24.2–32.4)	1,273 (766–1,781)
**Geography**			
Urban	6,107,062	25.4 (24.5–26.2)	663 (627–698)
Rural	1,511,825	28.2 (27.0–29.5)	871 (798–944)
**Self-reported health**			
Excellent	572,626	15.2 (13.3–17.2)	260 (228–292)
Very good	1,831,360	20.3 (19.3–21.4)	391 (361–421)
Good	2,357,029	24.7 (23.4–26.0)	589 (532–647)
Fair	1,893,376	37.3 (35.2–39.4)	1,180 (1,080–1,280)
Poor	941,100	47.9 (45.1–50.8)	2,255 (2,012–2,499)
**Functional characteristics**			
Blind/Difficulty seeing			
Yes	828,168	42.7 (38.7–46.8)	1,548 (1,341–1,754)
No	6,758,376	24.6 (23.9–25.4)	638 (607–670)
Difficulty concentrating			
Yes	1,362,936	50.9 (47.6–54.1)	1,944 (1,773–2,115)
No	6,175,049	23.2 (22.6–23.9)	566 (536–597)
Difficulty walking/climbing stairs			
Yes	3,189,778	47.3 (45.4–49.1)	1,735 (1,626–1,844)
No	4,388,844	19.4 (18.8–20.1)	389 (364–415)
Difficulty performing errands alone			
Yes	1,258,886	56.5 (52.9–60.0)	2,366 (2,127–2,604)
No	6,313,271	23.3 (22.6–24.0)	561 (532–590)
Difficulty dressing/bathing			
Yes	855,277	59.6 (55.3–63.8)	2,689 (2,365–3,014)
No	6,749,735	24.1 (23.4–24.8)	598 (568–627)
Any physical activity in past month			
Yes	4,900,264	23.3 (22.5–24.0)	574 (538–610)
No	2,707,832	32.5 (30.8–34.1)	1,013 (946–1,079)
**75–84 years**			
**Overall**	**4,424,372**	**28.5 (27.5–29.5)**	**707 (664–750)**
**Sex**			
Male	1,744,922	27.3 (25.6–28.9)	748 (670–826)
Female	2,671,039	29.4 (28.1–30.8)	679 (631–728)
**Race/Ethnicity** ^¶^			
White	3,660,879	29.8 (28.7–30.8)	742 (694–790)
Black	289,006	23.4 (18.7–28.8)	488 (397–579)
American Indian/Alaska Native	24,161	29.2 (22.0–37.7)	1,022 (657–1,386)
Asian/Pacific Islander	45,914	—**	—
Hispanic	267,023	24.8 (19.7–30.6)	498 (377–619)
Multiple/Other	62,832	31.1 (24.8–38.2)	—
**Geography**			
Urban	3,573,520	28.2 (27.0–29.4)	683 (634–732)
Rural	850,758	29.9 (28.3–31.6)	816 (731–901)
**Self-reported health**			
Excellent	305,524	17.9 (15.5–20.7)	328 (234–422)
Very good	1,031,504	23.5 (21.9–25.2)	443 (385–502)
Good	1,528,297	28.8 (26.9–30.8)	625 (569–682)
Fair	959,740	34.5 (32.0–37.0)	1,017 (892–1,143)
Poor	579,025	44.9 (40.5–49.3)	1,756 (1,454–2,058)
**Functional characteristics**			
Blind/Difficulty seeing			
Yes	482,311	39.8 (35.9–43.8)	1,461 (1,189–1,732)
No	3,929,486	27.6 (26.5–28.6)	643 (602–683)
Difficulty concentrating			
Yes	681,990	44.3 (40.8–47.8)	1,672 (1,417–1,927)
No	3,705,749	26.7 (25.6–27.8)	599 (560–638)
Difficulty walking/climbing stairs			
Yes	2,134,694	45.1 (42.9–47.4)	1,435 (1,314–1,556)
No	2,264,615	21.1 (20.1–22.2)	385 (353–416)
Difficulty performing errands alone			
Yes	814,654	50.3 (46.4–54.2)	1,906 (1,642–2,169)
No	3,590,020	25.9 (24.9–26.9)	566 (529–603)
Difficulty dressing/bathing			
Yes	486,255	58.0 (52.6–63.3)	2,423 (2,018–2,828)
No	3,927,919	26.8 (25.8–27.8)	608 (569–647)
Any physical activity in past month			
Yes	2,667,197	26.3 (25.0–27.6)	571 (525–617)
No	1,746,501	32.7 (31.0–34.5)	963 (874–1,052)
**≥85 years**			
**Overall**	**1,642,172**	**33.8 (31.8–35.9)**	**816 (719–913)**
**Sex**			
Male	660,820	35.7 (32.3–39.2)	931 (755–1,107)
Female	976,613	32.8 (30.3–35.4)	733 (621–846)
**Race/Ethnicity** ^¶^			
White	1,405,165	35.3 (33.2–37.5)	817 (737–897)
Black	79,686	26.0 (20.3–32.6)	580 (393–766)
American Indian/Alaska Native	8,547	—	—
Asian/Pacific Islander	10,034	—	—
Hispanic	86,926	39.8 (26.6–54.7)	—
Multiple/Other	17,663	35.0 (22.3–50.2)	789 (439–1,139)
**Geography**			
Urban	1,343,701	33.4 (31.1–35.8)	795 (682–908)
Rural	298,448	35.7 (31.8–39.9)	916 (773–1,059)
**Self-reported health**			
Excellent	96,407	21.6 (17.1–26.9)	373 (288–459)
Very good	338,642	28.0 (24.6–31.6)	544 (462–625)
Good	538,133	30.9 (27.7–34.3)	726 (549–902)
Fair	393,290	40.5 (35.5–45.7)	934 (791–1,078)
Poor	269,246	58.1 (51.6–64.4)	2,051 (1,418–2,685)
**Functional characteristics**			
Blind/Difficulty seeing			
Yes	301,101	44.7 (37.8–51.8)	1,435 (974–1,897)
No	1,326,118	31.9 (29.8–34.1)	714 (628–800)
Difficulty concentrating			
Yes	353,378	48.9 (41.0–56.8)	1,527 (1,091–1,962)
No	1,253,102	30.9 (29.0–32.8)	654 (601–707)
Difficulty walking/climbing stairs			
Yes	894,527	45.8 (42.4–49.2)	1,275 (1,094–1,457)
No	733,277	25.8 (23.2–28.5)	506 (395–617)
Difficulty performing errands alone			
Yes	504,470	49.5 (44.9–54.2)	1,319 (1,073–1,565)
No	1,114,674	29.4 (27.3–31.7)	679 (575–783)
Difficulty dressing/bathing			
Yes	243,067	56.9 (49.4–64.1)	1,991 (1,314–2,668)
No	1,390,938	31.5 (29.4–33.7)	701 (617–784)
Any physical activity in past month			
Yes	864,536	31.9 (29.2–34.7)	704 (584–824)
No	772,887	36.4 (33.4–39.5)	960 (800–1,119)

**Abbreviation**: CI = confidence interval.

* Weighted number of falls per 1,000 adults aged ≥65 years.

^†^ Weighted number of adults aged ≥65 years reporting at least one fall in the past year. Because of varying question-specific nonresponse, sample sizes might vary among questions.

^§^ Weighted percentage of adults aged ≥65 years reporting at least one fall in the past year.

^¶^ Whites, blacks, American Indians/Alaska Natives, Asians/Pacific Islanders, and others/unknown were non-Hispanic; Hispanics could be of any race.

** Dashes indicate sample size <50 or relative standard error >30%.

**TABLE 2:** Number of fall-related injuries, percentage of adults reporting a fall-related injury, and rates* of self-reported fall-related injuries in the past year among adults ≥65 years by age group and select characteristics (unweighted n = 142,591) — Behavioral Risk Factor Surveillance System, United States, 2018

Age group/Characteristic	No.^†^ reporting a fall-related injury	% of fall-related injuries^§^ (95% CI)	Rate* of fall-related injuries (95% CI)
**Total (all aged ≥65 years)**			
**Overall**	**5,051,046**	**10.2 (9.8–10.6)**	**170 (160–179)**
**Sex**			
Male	1,753,182	7.9 (7.4–8.6)	140 (125–155)
Female	3,285,921	11.9 (11.4–12.5)	193 (181–204)
**Race/Ethnicity** ^¶^			
White	3,927,593	10.2 (9.9–10.6)	170 (161–178)
Black	373,817	8.8 (7.1–10.8)	122 (99–144)
American Indian/Alaska Native	49,235	15.2 (11.4–19.9)	360 (183–536)
Asian/Pacific Islander	107,711	—**	90 (39–142)
Hispanic	422,695	11.5 (9.2–14.1)	192 (132–251)
Multiple/Other	73,334	11.3 (9.2–13.7)	—
**Geography**			
Urban	4,112,951	10.1 (9.6–10.6)	167 (157–178)
Rural	937,957	10.4 (9.8–11.1)	180 (161–199)
**Self-reported health**			
Excellent	322,006	5.4 (4.3–6.9)	65 (51–79)
Very good	972,529	6.7 (6.1–7.3)	81 (74–89)
Good	1,518,761	9.2 (8.5–9.8)	133 (122–145)
Fair	1,294,112	14.7 (13.6–15.9)	263 (238–289)
Poor	917,291	24.9 (23.0–26.9)	624 (535–713)
**Functional characteristics**			
Blind/Difficulty seeing			
Yes	742,101	19.6 (17.4–21.9)	436 (354–519)
No	4,281,945	9.4 (9.0–9.8)	147 (140–155)
Difficulty concentrating			
Yes	1,104,754	22.5 (20.6–24.6)	489 (425–552)
No	3,888,940	8.7 (8.4–9.1)	133 (125–141)
Difficulty walking/climbing stairs			
Yes	2,704,665	20.3 (19.2–21.3)	407 (376–438)
No	2,315,536	6.4 (6.0–6.8)	82 (76–88)
Difficulty performing errands alone			
Yes	1,318,985	27.3 (25.1–29.7)	587 (524–651)
No	3,693,519	8.3 (7.9–8.6)	124 (116–132)
Difficulty dressing/bathing			
Yes	833,239	31.2 (28.3–34.4)	724 (619–829)
No	4,198,368	8.9 (8.6–9.3)	138 (130–145)
Any physical activity in past month			
Yes	2,918,250	8.6 (8.1–9.1)	131 (121–140)
No	2,120,902	13.5 (12.7–14.3)	253 (232–274)
**65–74 years**			
**Overall**	**2,743,633**	**9.3 (8.8–9.9)**	**160 (148–171)**
**Sex**			
Male	958,537	6.9 (6.3–7.6)	123 (108–138)
Female	1,775,596	11.4 (10.7–12.2)	191 (175–208)
**Race/Ethnicity**			
White	1,999,023	9.0 (8.6–9.5)	155 (144–166)
Black	226,321	8.4 (6.9–10.2)	126 (100–153)
American Indian/Alaska Native	35,860	16.9 (11.9–23.9)	452 (191–714)
Asian/Pacific Islander	95,225	—	—
Hispanic	299,340	12.5 (9.5–16.3)	180 (136–224)
Multiple/Other	42,830	10.7 (8.6–13.3)	—
**Geography**			
Urban	511,500	9.3 (8.7–9.9)	160 (146–173)
Rural	2,232,054	9.6 (8.8–10.4)	161 (146–176)
**Self-reported health**			
Excellent	173,443	4.6 (3.1–6.8)	54 (35–73)
Very good	571,453	6.3 (5.6–7.1)	79 (69–89)
Good	744,975	7.8 (7.2–8.5)	116 (103–128)
Fair	765,642	15.1 (13.5–17.0)	276 (238–314)
Poor	477,503	24.5 (22.3–26.9)	649 (540–758)
**Functional characteristics**			
**Blind/Difficulty seeing**			
Yes	402,881	21.0 (17.5–24.9)	486 (366–605)
No	2,326,598	8.5 (8.0–9.0)	136 (128–145)
Difficulty concentrating			
Yes	642,512	24.2 (21.4–27.3)	529 (454–604)
No	2,064,220	7.8 (7.3–8.3)	121 (111–130)
Difficulty walking/climbing stairs			
Yes	1,408,428	21.0 (19.6–22.5)	452 (407–496)
No	1,324,451	5.9 (5.4–6.4)	73 (67–80)
Difficulty performing errands alone			
Yes	650,112	29.4 (26.0–33.0)	717 (600–834)
No	2,072,807	7.6 (7.2–8.1)	114 (106–121)
Difficulty dressing/bathing			
Yes	454,702	32.0 (28.4–35.9)	766 (633–899)
No	2,280,876	8.2 (7.7–8.7)	128 (118–138)
Any physical activity in past month			
Yes	1,620,337	7.7 (7.2–8.3)	121 (108–133)
No	1,118,474	13.4 (12.3–14.7)	258 (234–282)
**75–84 years**			
**Overall**	**1,634,953**	**10.6 (9.8–11.3)**	**170 (156–185)**
**Sex**			
Male	547,968	8.6 (7.4–9.9)	141 (118–164)
Female	1,085,428	12.0 (11.1–12.9)	192 (173–210)
**Race/Ethnicity**			
White	1,355,522	11.0 (10.3–11.8)	179 (164–195)
Black	115,601	9.3 (5.4–15.7)	112 (61–162)
American Indian/Alaska Native	7,702	9.4 (5.6–15.4)	179 (78–280)
Asian/Pacific Islander	9,402	—	—
Hispanic	90,085	8.4 (5.9–11.8)	135 (82–187)
Multiple/Other	21,322	10.6 (7.5–14.8)	173 (99–246)
**Geography**			
Urban	1,338,288	10.6 (9.7–11.5)	167 (151–183)
Rural	296,606	10.4 (9.5–11.5)	185 (149–222)
**Self-reported health**			
Excellent	112,211	6.6 (4.8–8.9)	80 (56–103)
Very good	301,804	6.9 (5.9–8.0)	82 (69–94)
Good	538,594	10.2 (8.7–11.8)	139 (120–157)
Fair	382,369	13.8 (12.3–15.4)	260 (220–300)
Poor	286,516	22.3 (19.2–25.7)	527 (408–647)
**Functional characteristics**			
Blind/Difficulty seeing			
Yes	190,201	15.8 (13.4–18.5)	338 (258–419)
No	1,440,008	10.1 (9.4–10.9)	156 (142–170)
Difficulty concentrating			
Yes	294,225	19.2 (16.6–22.2)	398 (324–472)
No	1,326,930	9.6 (8.8–10.4)	145 (131–159)
Difficulty walking/Climbing stairs			
Yes	889,083	18.9 (17.1–20.8)	360 (320–401)
No	731,862	6.8 (6.2–7.5)	86 (76–96)
Difficulty performing errands alone			
Yes	404,429	25.2 (21.3–29.4)	511 (432–591)
No	1,222,743	8.8 (8.2–9.5)	130 (118–143)
Difficulty dressing/Bathing			
Yes	248,895	30.1 (24.0–37.0)	636 (524–749)
No	1,379,549	9.4 (8.8–10.1)	144 (130–157)
Any physical activity in past month			
Yes	964,611	9.5 (8.6–10.5)	141 (125–157)
No	665,922	12.5 (11.4–13.7)	226 (198–254)
**≥85 years**			
**Overall**	**672,460**	**13.9 (12.5–15.4)**	**227 (179–276)**
**Sex**			
Male	246,677	13.4 (11.0–16.2)	265 (148–382)
Female	424,896	14.3 (12.7–16.1)	205 (175–236)
**Race/Ethnicity**			
White	573,048	14.5 (13.0–16.1)	222 (186–257)
Black	31,894	10.5 (7.1–15.2)	119 (74–164)
American Indian/Alaska Native	5,673	—	—
Asian/Pacific Islander	3,084	—	—
Hispanic	33,270	—	—
Multiple/Other	9,182	—	—
**Geography**			
Urban	542,610	13.6 (12.1–15.2)	216 (163–268)
Rural	129,850	15.6 (12.1–19.8)	283 (155–410)
**Self-reported health**			
Excellent	36,352	8.2 (5.3–12.3)	96 (59–133)
Very good	99,273	8.2 (6.5–10.4)	100 (77–123)
Good	235,192	13.6 (11.4–16.1)	216 (150–282)
Fair	146,101	15.1 (12.5–18.2)	203 (165–241)
Poor	153,272	33.4 (26.5–41.1)	788 (367–1210)
**Functional characteristics**			
Blind/Difficulty seeing			
Yes	149,020	22.4 (17.6–28.0)	—
No	515,339	12.5 (11.1–14.0)	187 (154–221)
Difficulty concentrating			
Yes	168,017	23.4 (17.8–30.2)	532 (234–831)
No	497,790	12.3 (11.1–13.7)	174 (150–198)
Difficulty walking/climbing stairs			
Yes	407,155	21.0 (18.5–23.7)	366 (261–470)
No	259,223	9.1 (7.6–10.9)	133 (91–174)
Difficulty performing errands alone			
Yes	264,445	26.2 (22.1–30.7)	424 (311–536)
No	397,969	10.5 (9.3–11.9)	174 (120–227)
Difficulty dressing/bathing			
Yes	129,643	30.9 (24.5–38.2)	—
No	537,943	12.2 (10.9–13.7)	176 (144–207)
Any physical activity in past month			
Yes	333,302	12.3 (10.5–14.4)	171 (142–201)
No	336,507	15.9 (13.8–18.3)	298 (194–403)

**Abbreviation:** CI = confidence interval.

* Weighted number of fall-related injuries per 1,000 older adults.

^†^ Weighted number of adults aged ≥65 years reporting at least one fall-related injury in the past year. Because of varying question-specific nonresponse, sample sizes might vary among questions.

^§^ Weighted percentage of older adults reporting at least one fall-related injury in the past year.

^¶^ Whites, blacks, American Indians/Alaska Natives, Asians/Pacific Islanders, and others/unknown were non-Hispanic; Hispanics could be of any race.

** Dashes indicate sample size <50 or relative standard error >30%.

Among states in which falls and fall injuries were consistently reported across years (excluding Michigan, Oregon, and Wisconsin where data in 2012 were reported differently than in other years), the percentage of those older adults reporting a fall increased from 27.9% in 2012 to 29.6% in 2016 (p<0.001) and decreased to 27.4% in 2018 (p<0.001) (Figure). The rates of falls and fall-related injuries and the percentages of older adults reporting a fall-related injury did not significantly change from 2012 to 2018.

**FIGURE:**
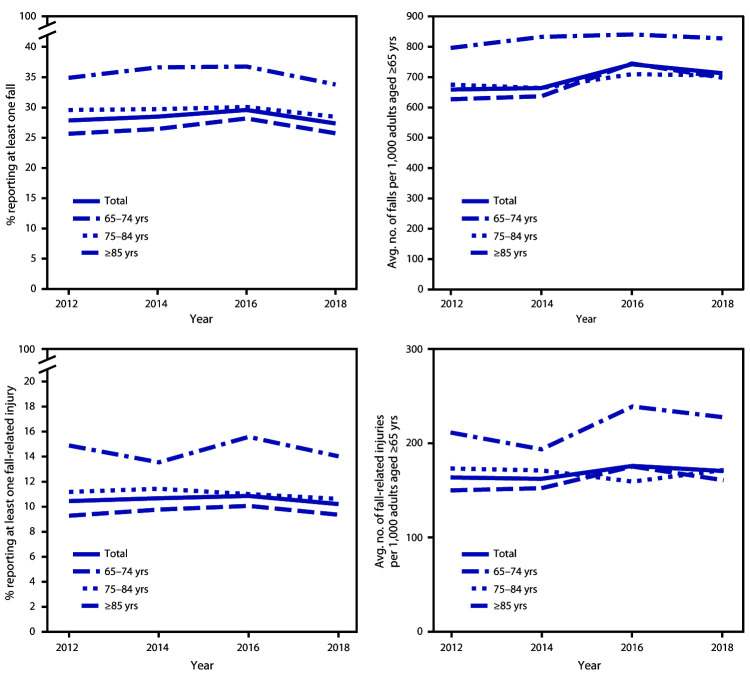
Percentages and rates of self-reported falls and fall-related injuries among adults aged ≥65 years, by age group — Behavioral Risk Factor Surveillance System, United States,* 2012–2018 * Data from Michigan, Oregon, and Wisconsin were omitted because of the difference in the way these states collected information about falls during 2012, compared with the rest of the states.

## Discussion

The percentage of older adults reporting a fall increased from 2012 to 2016, followed by a modest decline from 2016 to 2018. Although statistically significant, these changes were small. Even with this decrease in 2018, older adults reported 35.6 million falls. Among those falls, 8.4 million resulted in an injury that limited regular activities for at least a day or resulted in a medical visit. In the United States, health care spending on older adult falls has been approximately $50 billion annually (*3*). In 2018, approximately 52 million adults were aged ≥65 years^§^ by 2030, this number will increase to approximately 73 million.^¶^ Despite no significant changes in the rate of fall-related injuries from 2012 to 2018, the number of fall-related injuries and health care costs can be expected to increase as the proportion of older adults in the United States grows.

Adults aged ≥85 years were more likely to report a fall or fall-related injury in the preceding year than were those aged <85 years. Currently, adults aged ≥85 years account for <2% of the population; by 2050 this proportion is projected to increase to 5%. Many fall risk factors increase with age, including chronic health conditions related to falls, increased use of medications, and functional decline (*4*). More research is needed to determine how fall risk factors differ among persons aged ≥85 years and to identify targeted interventions that could adequately address the needs of these adults.

The findings in this report are subject to at least five limitations. First, because BRFSS data are self-reported, they are subject to recall bias, especially for falls that did not result in injury or that occurred several months before the survey (*5*). Second, this survey is cross-sectional. Although functional abilities, health status, and physical activity were all associated with falls and fall-related injuries, it is not possible to determine whether these factors preceded the fall or resulted from a fall. Third, BRFSS does not include older adults living in nursing homes, which might have led to an underestimation of falls and fall-related injuries, especially among adults aged ≥85 years (*6*). Fourth, the response rate (median response rate of 49.9%) could result in non-response bias, however BRFSS data are weighted to adjust for some of this bias. Finally, the results of the trend analyses were derived from only four time points. Future analyses with more time points might describe these trends with more certainty.

Regardless of age group, higher percentages of older adults who reported no physical activity in the past month or reported difficulty with one or more functional characteristics (difficulty walking up or down stairs, dressing and bathing, and performing errands alone) reported falls and fall-related injuries. These risk factors are frequently modifiable suggesting that, regardless of age, many falls might be prevented. Older adults of any age can, together with their health care providers, take steps to reduce their risk for falls. CDC created the Stopping Elderly Accidents, Deaths & Injuries (STEADI) initiative, which offers tools and resources for health care providers to screen their older patients for fall risk, assess modifiable fall risk factors, and to intervene with evidence-based fall prevention interventions (https://www.cdc.gov/steadi). These include medication management, vision screening, home modifications, referral to physical therapists who can address problems with gait, strength, and balance, and referral to effective community-based fall prevention programs. As the proportion of older adults living in the United States continues to grow, so too will the number of falls and fall-related injuries. However, many of these falls are preventable. To help keep older adults living independently and injury-free, reducing fall risk and fall-related injuries is essential.

**Summary**

**What is already known about this topic?**

Falls are the leading cause of injury among adults aged ≥65 years, who in 2014 experienced an estimated 29 million falls, resulting in 7 million fall-related injuries.

**What is added by this report?**

In 2018, 27.5% of adults aged ≥65 years reported at least one fall in the past year (35.6 million falls) and 10.2% reported a fall-related injury (8.4 million fall-related injuries). From 2012 to 2016, the percentages of these adults reporting a fall increased, and from 2016 to 2018, the percentages decreased.

**What are the implications for public health practice?**

Falls and fall-related injuries are highly prevalent but are preventable. Health care providers play a crucial role and can help older adults reduce their risk for falls.

## Footnotes

* www.cdc.gov/injury/wisqars.

† https://www.cdc.gov/brfss.

§ https://www2.census.gov/programs-surveys/popest/tables/2010-2018/counties/asrh/PEPAGESEX.pdf.

¶ https://www.census.gov/data/tables/2017/demo/popproj/2017-summary-tables.html.
