# *Erratum:* Vol. 69, No. 27

**DOI:** 10.15585/mmwr.mm7218a6

**Published:** 2023-05-05

In the report “Trends in Nonfatal Falls and Fall-Related Injuries Among Adults Aged ≥65 Years — United States 2012–2018,” on page 876, in Table 1, for the category “Total, all aged ≥65 years,” among Black persons, the number reporting a fall should have read **957,303**, and among American Indian or Alaska Native persons, should have read **104,915**. On page 878, in Table 2, for the category “65–74 years,” among American Indian or Alaska Native persons, the percentage of fall-related injuries and corresponding 95% CI should have read **16.9 (11.9–23.3)**.

